# Antibiotic use, knowledge and health literacy among the general population in Berlin, Germany and its surrounding rural areas

**DOI:** 10.1371/journal.pone.0193336

**Published:** 2018-02-23

**Authors:** Florian Salm, Clemens Ernsting, Adelheid Kuhlmey, Melanie Kanzler, Petra Gastmeier, Paul Gellert

**Affiliations:** 1 Institute of Hygiene and Environmental Medicine, Charité–Universitätsmedizin, Berlin, Germany; 2 Institute for Infection Prevention and Hospital Epidemiology, Medical Center–University of Freiburg, Faculty of Medicine, University of Freiburg, Freiburg, Germany; 3 Institute of Medical Sociology, Charité–Universitätsmedizin, Berlin, Germany; 4 German Protestant Hospital Association, DEKV, Berlin, Germany; Augusta University, UNITED STATES

## Abstract

**Objectives:**

Knowledge concerning antibiotic use in the general population is insufficient. The way health literacy is related to antibiotic use aside from knowledge needs further investigation. Our aim was to compare the levels of knowledge of antibiotics and health literacy in individuals who had taken antibiotics in recent years compared with those who not had taken antibiotics.

**Methods:**

A population-based cross-sectional survey of 2,000 individuals aged 35 and older from Berlin, Germany and its surrounding rural and suburban areas (response rate 59%) with strata urban/rural, sex, age, and education. Computer-assisted personal interviews were conducted by external, trained interviewers during home visits. Knowledge, health literacy, and antibiotic use were assessed using standardized questionnaires.

**Results:**

In all, 33.3% (666/2,000) of the participants indicated having had an antimicrobial therapy during the previous 12 months. Adjusting for sex, age, educational level and health literacy, individuals with four correct answers regarding antibiotics were 1.70 times and those with three correct answers 1.94 more likely to have had a history of recent antibiotic use than those who did not have any correct answers. Individuals with sufficient health literacy were 0.57 times less likely to have had a recent history of antibiotic use than individuals with insufficient health literacy.

**Conclusion:**

Patients who have used antibiotics might have more knowledge as a result of their recent involvement with the topic of antibiotic use; health literacy may be a preventive mechanism to use antibiotics more critically. Besides improving the health knowledge of the general population and of vulnerable groups such as patients with low levels of health literacy, intervention strategies should focus on providers as well.

## Introduction

The rise of drug-resistant organisms poses a challenge for modern medicine [1]. Antimicrobial resistance is a natural phenomenon [2], but it exacerbated with antimicrobial exposure [3,4]. In Germany, about 75% of all antibiotics prescribed are used in ambulatory care [5], most of them for respiratory tract infections, which are caused predominantly by viruses, and for urinary tract infections [6,7]. In regions with higher consumption of antibiotics in outpatient care, there are higher rates of resistant bacteria [8]. Antibiotic use in primary care has remained relatively stable in recent years, with an decrease in the use of the basic penicillins and an increase in the use of reserve antibiotics [9,10]. Many primary care physicians feel pressured by their patients to prescribe antibiotics for infections which do not necessarily require antibiotics–i.e., pressured by patients who are not fully aware of the difference between viral and bacterial infections, for example, influenza, colds or sore throat [11,12]. Knowledge of the mode of action of antibiotics in the general population is insufficient in Europe as much as it is in Germany. This may have detrimental effects on prescription and intake routines [13,14]. Furthermore, the benefits of antimicrobial therapy are often inflated in comparison to their disadvantages for patients with an acute infection. “Antibiotics might not make me better, but I should take them just in case” [15]. Over the last years, health literacy has gained importance in the literature as a key factor in the promotion of health and for coping with illness [16,17]. Low health literacy has been shown to be associated with inappropriate use of the health care system [18]. It should be noted that health literacy differs from knowledge [17]. Health literacy describes the ability to understand and critically evaluate health information and to make health-related decisions [19]. Health literacy influences patient-provider relationship, self-care and the use of the health care system [20].

Moreover, research has shown that knowledge alone might not be sufficient to change health-related behavior. However, although competencies like health literacy may be crucial for efficiently managing health and illness by patients, research in the field of antibiotic use is sparse [21]. Thus, the aim of the study was to investigate the history of antibiotic use in the general population and to characterize consumers in terms of health literacy and knowledge.

## Methods

A population-based sample of 2,000 individuals from Berlin, Germany and its surrounding rural and suburban areas participated in this cross-sectional survey (Pfizer Monitor II). The Pfizer Monitor is part of a series of ad hoc surveys conducted in Germany related to topics of health knowledge and health literacy (conducted by Pfizer Deutschland GmbH in cooperation with Charité–Universitätsmedizin Berlin). The present study draws on data from the second wave of the Pfizer Monitor, which genuinely focused on health knowledge and health literacy regarding antibiotics and antimicrobial resistance. The sample size was opportunistic; a sensitivity analysis showed that the sample size allows for detecting a small mean difference of Cohen’s d of 0.16 (Sample size 666/2000 with a history of antibiotics; 1-β = .95, α = .05, two-sided, t-test using GPower 3.1.9.2).

### Recruitment process

In the pre-recruitment period the interviewers were informed about the upcoming study and the target group description according to socioeconomic factors required for representativeness. Afterwards, the interviewers had three to four days for the pre-recruitment process. Interviewers were free to choose the type and manner of recruitment. This was done door-to-door, in public places, with colleagues, or at the workplace. Family members and several members of the same profession were not allowed to participate. After the pre-recruitment period, the interviewers informed the coordination office of the opinion research institute about how many interviews can be carried and the socioeconomic data of the potential participants. Subsequently, the interviewer receives confirmation of the persons to be interviewed. Participants were interviewed in October and November 2016 by visiting their homes. All participants signed an informed consent. Stratified sampling was used based on the strata urban/rural, sex, age, and education, which are reported demographic factors associated with health literacy [20]. Participants had to satisfy the following inclusion criteria: a) a resident of Germany, b) sufficient German language skills, and c) 35 years of age or older. Residency was defined by asking the participant whether they now live in Germany. The cut-off was chosen to be in line with the German Ageing Survey (DEAS), a representative cohorts study [22] There were no exclusion criteria. After the first contact, appointments for home visits were made. There was a response rate of 59%, thus, in order to reach 2,000 participants, 3,390 were asked to take part. Computer-assisted personal interviews (CAPI) were conducted by external, trained interviewers during home visits. Of the individuals interviewed, 8% declined to complete the survey and their data was subsequently deleted.

### Antibiotic use and knowledge

Participants’ antibiotic use was assessed in accordance with the WHO [23] survey by asking them whether they had taken antibiotics during the previous year (yes/no).

Knowledge about antibiotics was assessed by asking participants four questions (Q1-Q4, see Table 1). Two questions (Q1, Q2) were taken from the systematic review of Gualano et al. [14] while the other two questions (Q3, Q4) were taken from the multi-country public awareness survey conducted by the World Health Organization (WHO) [23].

**Table 1:** Questionnaire statements. A cross-sectional survey of a population-based sample of 2,000 individuals from Berlin and its surrounding rural and suburban areas.

Knowledge		Correct Responses
Q1.	“Antibiotics can treat **bacterial** infections.” (True)	86.9% (1,738)
Q2.	“Antibiotics can treat **viral** infections.” (False)	68.7% (1,373)
Q3.	“Do you think the **common cold** can be treated with antibiotics?” (False)	60.2% (1,203)
Q4.	“Do you think **flu** can be treated with antibiotics?” (False)	58.7% (1,173)
**Statements**		
Q5.	“It’s okay to use antibiotics that were prescribed to a friend or family member, as long as they were used to treat the same illness.” (False)	71.6% (1,432)
Q6.	“Antibiotic resistance occurs when your body becomes resistant to antibiotics and they no longer work as well.” (False)	28.7% (574)

*Note*. Number of correct answers calculated by the Question Q1-Q4. Q1 and Q2 from Gualano et al. [14]; Q3-Q6 from WHO-Survey [23].

We generated a score of correct answers about the use of antibiotics indicated for viral infections, bacterial infections, influenza, and colds ranging from zero to four correct answers. The score accounted for knowledge of various facts. First, unlike viruses, bacteria can be treated with antibiotics. Second, the pathogenesis of an infection is important–common colds and influenza are caused by a virus. Often some of these facts are known, some are not [14,24].

Finally, participants had to indicate whether statements (Q5, Q6) were true or false. These statements were taken from the multi-country public awareness survey conducted by the WHO [23].

### Antibiotic use and health literacy

Health literacy was assessed using the 16-item short-form of the HLS-EU-Q instrument. This scale measures the capability to access, understand, appraise and apply health information [25]. Example items include “On a scale from very easy to very difficult, how easy would you say it is to …find information about symptoms of illnesses that concern you?” or “…use information the doctor gives you to make decisions about your illness?” In accordance with Sørensen et al. [25], individuals have been classified as having inadequate (0 to 8 points), problematic (9 to 12 points), or sufficient understanding (above 12 to 16 points).

### Educational level

In accordance with the International Standard Classification of Education (ISCED), educational level was divided into three categories: “no or basic qualifications (ISCED 1–2)”, “vocational qualification (ISCED 3–4),” and “degree (ISCED 5–6)”. The HLS-EU-Q16 is an established instrument to measure health literacy and has been validated and frequently used in different German samples [26–28].

### Statistical analysis

**Fig 1:**
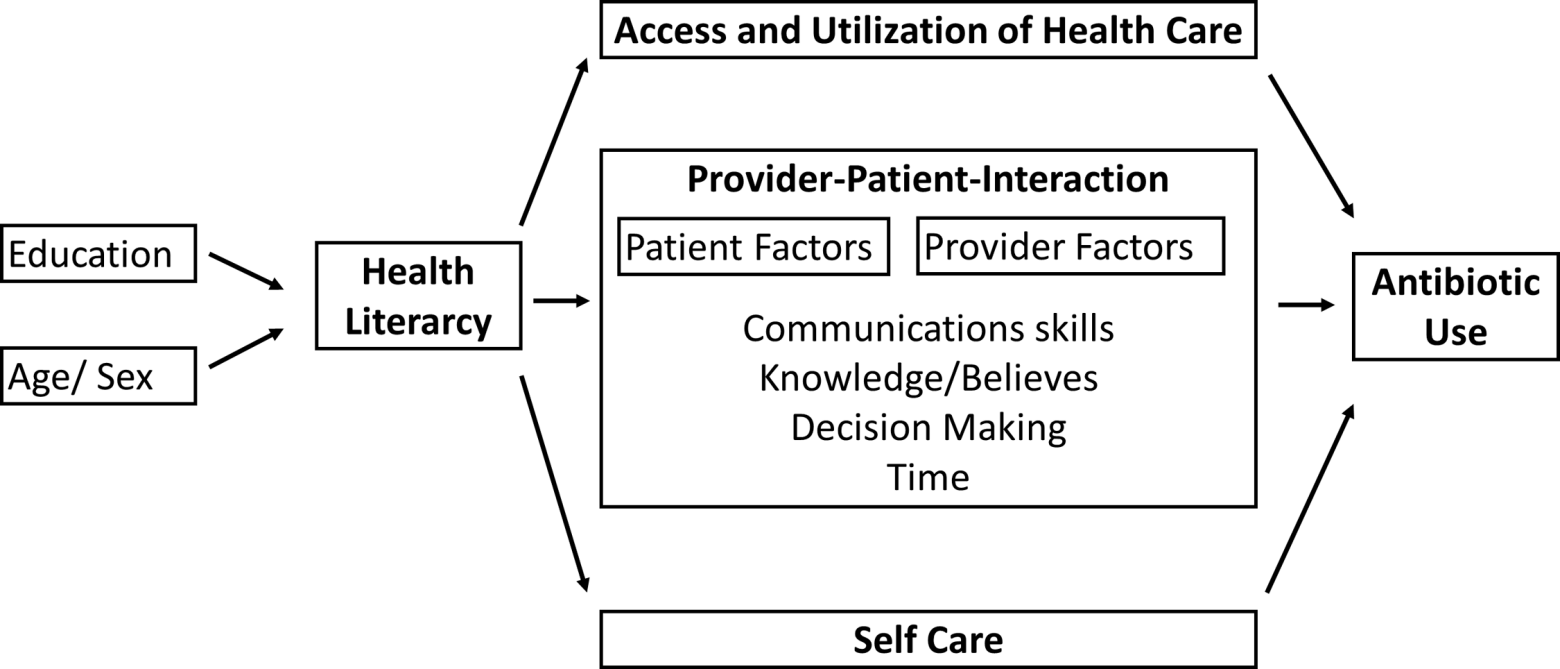
Causal pathways between health literacy and antibiotic use. Figure adapted from Paasche-Orlow and Wolf, 2007 [20].

Binary logistic regression with antibiotics taken during the previous twelve months as outcome was conducted, with sex, age, educational level, knowledge regarding antibiotics and health literacy as covariates. Significance level of p = 0.05 was used. All analyses were performed using IBM SPSS statistics, version 23. A theoretical model showing the relationships between variables can be found in Fig 1.

Ethical approval was not obtained because after a telephone consultation the analysis was classified as secondary data analysis by the head of the local ethics committee office. In Germany, secondary data analyses do not require ethical approval [27]. All data were collected and analyzed anonymously.

## Results

The sample was comprised of 48.9% (977/2,000) women with a mean age of 59 (range 25–81 years of age; see Table 2). A total of 33.3% (666/2,000) of the participants had an antimicrobial therapy during the previous 12 months and 88.7% said that they received advice from a doctor or pharmacist on how to take the prescribed antibiotics; 22.9% agreed with the statement that a friend’s antibiotics could be taken as long as they were used to treat the same illness (Q5). In all, 63.7% of the participants thought that the human body can become resistant to antibiotics (Q6).

**Table 2:** Health literacy and the use of antibiotics within the previous 12 months. Cross-sectional survey of a population-based sample of 2,000 individuals from Berlin and its surrounding rural and suburban areas.

		Antibiotics taken within the previous year	
	Total	No (in %)	Yes (in %)
Total	2,000	1,334 (66.7)	666 (33.3)
Sex (men)	977 (48.9)	664 (68.0)	313 (32.0)
Age			
35–44	453 (22.7)	335 (73.0)	118 (26.0)
34–54	505 (25.3)	331 (65.5)	174 (34.5)
55–64	415 (20.8)	277 (66.7)	138 (33.3)
65–74	356 (17.8)	221 (62.1)	135 (37.9)
75+	271 (13.6)	170 (62.7)	101 (37.3)
Educational level			
Basic	296 (14.8)	176 (59.5)	120 (40.5)
Vocational	1,407 (70.4)	941 (66.9)	466 (33.1)
Degree	297 (14.9)	217 (73.1)	80 (26.9)
Knowledge*			
0	79 (4.0)	58 (73.4)	21 (26.6)
1	324 (16.2)	233 (71.9)	91 (28.1)
2	382 (19.1)	255 (66.8)	127 (33.2)
3	461 (23.1)	279 (60.5)	182 (39.5)
4	754 (37.7)	509 (67.5)	245 (32.5)
Health Literacy			
Inadequate	294 (14.7)	175 (59.5)	119 (40.5)
Problematic	489 (24.5)	284 (58.1)	205 (41.9)
Sufficient	1,217 (60.9)	875 (71.9)	342 (28.1)

*Note*. *number of correct answers, calculated by the Question Q1-Q4

**Fig 2:**
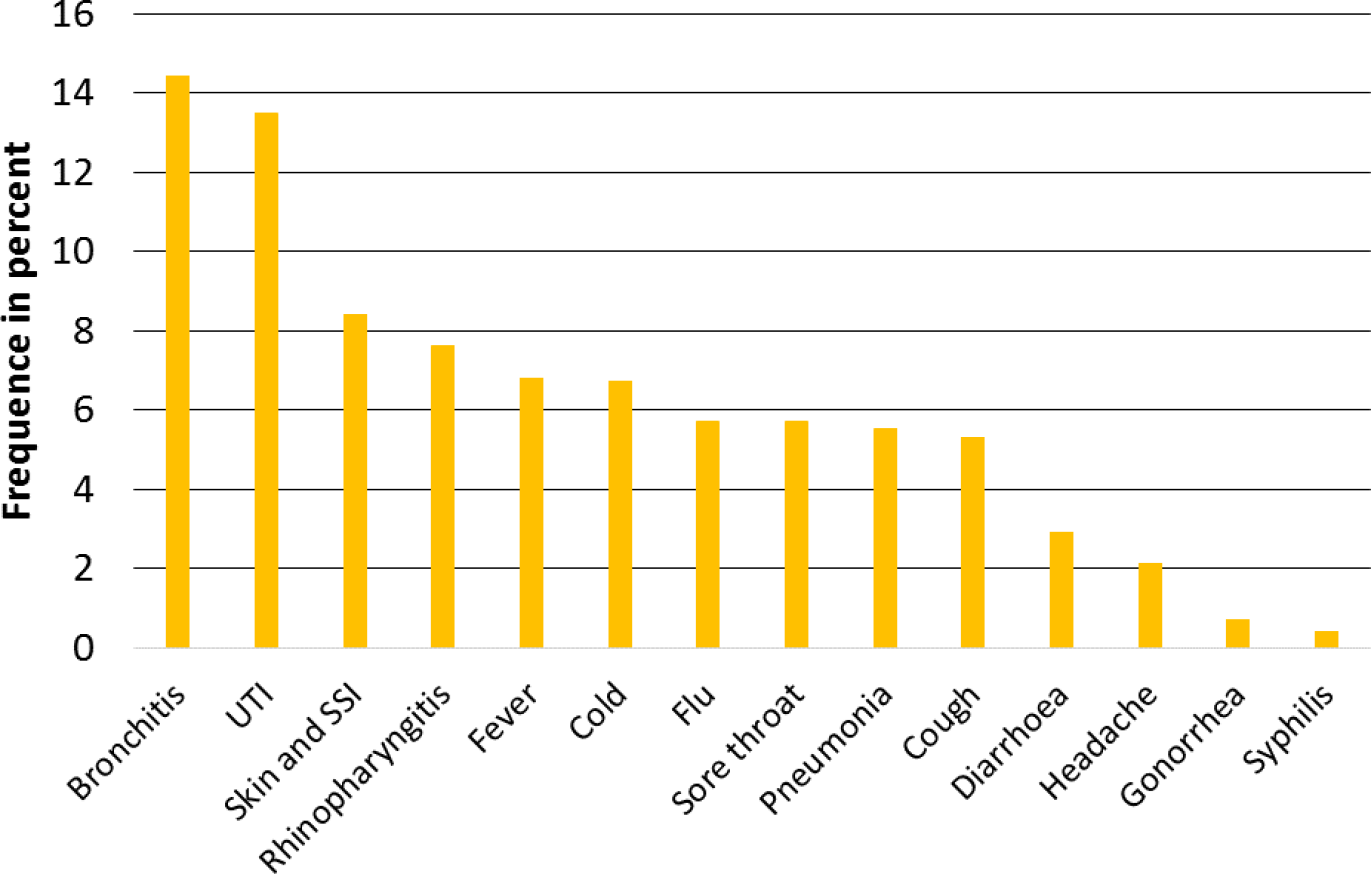
Main reasons for having antimicrobial therapy. Cross-sectional survey of a population-based sample of 2,000 individuals from Berlin and its surrounding rural and suburban areas. Note. UTI, urinary tract infection; SSI, surgical site infection. 12.8% noted for other reasons and 7.9% I don´t know.

Fig 2 shows the reasons for taking an antibiotic.

When adjusting for sex, age and educational level and health literacy, individuals with four correct answers about antibiotics were 1.70 times more likely to have had a history of recent use of antibiotics than those who did not have any correct answers and those with three correct answers were 1.94 more likely to have had such a history (see Table 3). In the multivariate model, individuals with sufficient health literacy were 0.57 times less likely to have had a recent history of antibiotics use than individuals with insufficient health literacy.

**Table 3:** Multivariate associations with history of antibiotics. A cross-sectional survey of a population-based sample of 2,000 individuals from Berlin and its surrounding rural and suburban areas.

	Logistic Regression Model			
	OR	UL95%CI	LL95%CI	P
Sex (men)	0.92	0.76	1.11	.373
Age				.083
35–44	Ref.			
45–54	1.47	1.11	1.95	.008
55–64	1.29	0.95	1.74	.098
65–74	1.44	1.06	1.98	.021
75+	1.30	0.92	1.83	.14
Educational level				.010
Basic	Ref.			
Vocational	0.79	0.60	1.03	.085
Degree	0.57	0.40	0.82	.002
Knowledge				<.001
None correct	Ref.			
One correct	1.03	0.58	1.81	.926
Two correct	1.49	0.86	2.60	.157
Three correct	1.94	1.13	3.35	.017
Four correct	1.70	0.99	2.92	.053
Health literacy				<.001
Inadequate	Ref.			
Problematic	1.06	0.78	1.44	.699
Sufficient	0.57	0.43	0.76	<.001

Note. OR = Odds Ratio, LL 95%CI/ UL 95%CI Upper and lower limit 95% confidence intervals

Age was a factor influencing antibiotic use. Younger participants (age <44 years) were less likely to have taken antibiotics during the previous 12 months.

## Discussion

We investigated knowledge of antibiotics and their actual use, as well as the health literacy of the general population in Berlin and its surrounding areas.

In this study, individuals who had taken antibiotics during the previous year showed better knowledge of antibiotics than those who reported not having taken any antibiotics. Conversely, individuals who had not taken antibiotics during the previous year showed better health literacy than participants who had taken antibiotics during the previous twelve months. We found three of four correct answers (Q1-Q4) had the highest risk for an antibiotic exposure in the last 12 months. It appears that patients with exposure gain sufficient yet not comprehensive knowledge through their antibiotics use experience. This is in line with findings from the Special Eurobarometer that showed that patients who have taken antibiotics within the last year were more likely to have fragmentary knowledge about the effects of antibiotics [28].

Many antibiotic campaigns address the awareness and knowledge of antimicrobial resistance [29,30]. Our findings concerning this knowledge may reflect the phenomenon that affected people have more knowledge about what matters to them, for example people with severe pain [31]. Shallcross et al. found that individuals who had taken antibiotics during the previous year were more likely to have taken antibiotics during the previous three years [32]. However, it is still not fully understood why knowledge is higher in the group with a recent history of antibiotic use. Nevertheless, the majority agreed with the statement “antibiotic resistance occurs when your body becomes resistant to antibiotics and they no longer work as well.” Knowledge of antibiotics seems to be fragmentary in the general population.

Approaches sensitive to health literacy can improve the knowledge of the general population [33]. In addition to increasing awareness and knowledge of antibiotics, competencies such as health literacy should be targets of intervention, especially in the primary health care setting. When people are ill, they tend more to take risks and to accept side effects, even if they know the expected benefits are low [15].

One-third (33%) of the participants had taken antibiotics during the previous 12 months. This rate of self-reported antibiotic use is higher than rates of 23% reported in the Eurobarometer for Germany [28] and 22% in a representative survey of the German general population by Schneider et al. [13] although the questions were comparable in all investigations. Compared with other regions in Germany, Berlin, in particular its surrounding areas, has the lowest antibiotic prescription rates of daily defined doses per patient in ambulatory care [5], although we found higher rates of self-reported antibiotic history there. This may be partly due to the time of the assessment interval since Eurobarometer and the study by Schneider et al. were conducted in spring and the present investigation was conducted in fall. Nonetheless, the rate is much lower than the multi-country average of 77% self-reported antibiotic use of the WHO [23].

### Limitations

There were several limitations on our findings. A limitation due to the study design is that the influence of the prescriber was not measured. Provider-patient interaction is influenced by patient factor (e.g. knowledge and health literacy) and by provider factors (e.g. time, communication skills). The present study primarily focused on the patient’s perspective. Based on the results of these findings, future studies should further investigate provider-patient interaction that may be influenced by patient factors (e.g., knowledge and health literacy) and by provider factors (e.g., time, communication skills) as studies have shown [18,20,33]. For instance, patients with specific knowledge on antibiotics but rather limited comprehensive health literacy skills that include critical evaluation new information and communication self-efficacy may require other provider skills compared with patients that have sufficient health literacy but are not yet well-informed about the specific topic of antibiotics use.

Based on the results of this investigation, following question arises: how is the provider-patient interaction effected by health literacy and knowledge, especially of moderate patient on the outcome antimicrobial prescription? Furthermore, it remains unclear if the antibiotic use was appropriate.

We defined health literacy as the general perceived ability to acquire, evaluate and act on health information, thus, a self-report instrument was suitable. Although this conceptualization of health literacy as an individual control belief is an important driver of human behavior [34], they may over or underestimate actual health literacy skills.

In the present study, we focused on a small set of very specific knowledge concerning the use of antibiotics that we thought would be the essential knowledge that to know would make a difference in prescription routines at scale (i.e., differentiate between cold, flu, virus, and bacteria). Nonetheless, future investigations should elaborate the full complexity of knowledge and its influence on decision making in antibiotics. The development of an internationally validated assessment tool for antibiotics knowledge and decision making would be desirable. Furthermore, the influence of increased age on increasing consumption of antibiotics (see Table 3), which has also been described by other researchers [9,35], should be the focus of other approaches to reducing antibiotic use. While the oldest old over 80 years usually show decreased levels of health knowledge and health literacy yet increased need for antibiotics, younger cohorts of older adults often show good knowledge and health literacy [36,37]. Interventions should specifically balance the need for antibiotics with clear age-tailored communication strategies in cases when antibiotics are clearly inappropriate.

A strength of the study is the large number of participants and the manner in which the interviews were conducted as a personal face-to-face interview.

In conclusion, in our study, participants who had taken antibiotics in the previous year had higher knowledge of antibiotics, but health literacy was lower since those with sufficient health literacy were less likely to have used antibiotics recently. Besides improving the health knowledge of the general population and of vulnerable groups such as patients with low levels of health literacy, intervention strategies should focus on providers as well. Concerning health education of the public, societies need more effective ways of building health literacy along with building literacy and educating the public. Health and health literacy courses in schools may have an impact at scale. Concerning providers, providers are influenced by patients’ expectations. Interventions should enable providers to address health literacy challenges in clinical encounters. Effective very brief interventions that can be delivered in one or in five minutes by health care professionals, which have been found to be effective for a variety of health-related behaviors may be adapted for the case of antibiotics use [38,39]. These very brief intervention have the possibility to achieve significant public health impact. For antibiotics prescription, a study impressively showed that a brief behavioral intervention among primary care practices was effective in decreasing rates of inappropriate antibiotic prescribing for acute respiratory tract infections [40].

## Supporting information

S1 Dataset Legend and syntax. This file contains the legend of the dataset and the SPSS syntax of the analysis.
(DOCX)

S2 Dataset This file contains all the data used. (XLSX)
