# Supplementary material for: Antibiotic use, knowledge and health literacy among the general population in Berlin, Germany and its surrounding rural areas
Source: PLoS One. 2018 Feb 23;13(2):e0193336. doi: 10.1371/journal.pone.0193336 (PMC5825110; doi:10.1371/journal.pone.0193336)
Supplement: S1 Dataset — This file contains the legend of the dataset and the SPSS syntax of the analysis. (DOCX) [file pone.0193336.s001.docx]

Dataset_Names

|  | **Socio-economic** |
| --- | --- |
| S1 | Age (in years) |
| age_cat | Age categorized (0= 35-44 [years], 1= 45-54; 2=55-64; 3=65-74; 5=75+) |
| men | Sex (0=male; 1=female) |
| EDU | Educational level (1= Basic, 2=Vocational, 3= Degree) |
|  | **Knowledge** |
| B2A3 | “Antibiotics can treat bacterial infections.” [True] (0=not correct, 1=correct response) |
| Q2. | “Antibiotics can treat viral infections.” [False] (0=not correct, 1=correct response) |
| B1A4_neu | “Do you think the common cold can be treated with antibiotics?“ [False]  (0=not correct, 1=correct response) |
| B1A5_neu | “Do you think flu can be treated with antibiotics?“ [False] (0=not correct, 1=correct response) |
| Wissen4 | SUM(B1A4_neu, B1A5_neu, B2A3_neu, B2A4_neu) |
|  | **Statements** |
| B2A1_neu | “It’s okay to use antibiotics that were prescribed to a friend or family member, as long as they were used to treat the same illness.” [False] (0=not correct, 1=correc response) |
| B3A1_neu | “Antibiotic resistance occurs when your body becomes resistant to antibiotics and they no longer work as well.” [False] (0=not correct, 1=correct response) |
|  | **Health Literacy** |
| HLSrr | Sum of EUROHIS-QoL-16 (min=1; max=16) |
| HLScat | Health literacy categorized (1=inadequate, 2=problematic, 3=sufficient) |
| antibioticslastyear | Antibiotic exposure within the last 12 months (0=not correct, 1=correct) |

SPSS-Syntax:

******SPSS SYNTAX - HEALTH LITERACY AND KNOWLEDGE REGADRING ANTIBIOTICS *******

**** MODELLTESTS ******

CROSSTABS

/TABLES= wissen4 BY antibioticslastyear

/FORMAT=AVALUE TABLES /STATISTICS=CHISQ /CELLS=COUNT EXPECTED ROW COLUMN TOTAL PROP /COUNT ROUND CELL.

CROSSTABS

/TABLES= HLScat BY antibioticslastyear

/FORMAT=AVALUE TABLES /STATISTICS=CHISQ /CELLS=COUNT EXPECTED ROW COLUMN TOTAL PROP /COUNT ROUND CELL.

CROSSTABS

/TABLES= age_cat BY antibioticslastyear

/FORMAT=AVALUE TABLES /STATISTICS=CHISQ /CELLS=COUNT EXPECTED ROW COLUMN TOTAL PROP /COUNT ROUND CELL.

**REGRESSION MODEL. REFERENCE VALUE =FIRST**.

LOGISTIC REGRESSION VARIABLES antibioticslastyear

/METHOD=ENTER age_cat men edu

/METHOD=ENTER age_cat men edu wissen4

/METHOD=ENTER age_cat men edu wissen4 HLScat

/CONTRAST (HLScat)=Indicator(1)

/CONTRAST (wissen4)=Indicator(1)

/CONTRAST (edu)=Indicator(1)

/CONTRAST (age_cat)=Indicator(1)

/PRINT=ALL

/CRITERIA=PIN(.05) POUT(.10) ITERATE(20) CUT(.5).

***TABLE 1**.

FREQUENCIES VARIABLES=age_cat men edu wissen4 HLScat

/ORDER=ANALYSIS.

SORT CASES BY antibioticslastyear.

SPLIT FILE SEPARATE BY antibioticslastyear.

FREQUENCIES VARIABLES=age_cat men edu wissen4 HLScat

/ORDER=ANALYSIS.

SPLIT FILE OFF.

FILTER OFF.

USE ALL.

EXECUTE.
